# Editorial: All roads lead to Rome: Harnessing thalamic neuromodulation for difficult-to-treat neurological disorders

**DOI:** 10.3389/fnhum.2023.1155605

**Published:** 2023-02-14

**Authors:** Glenn D. R. Watson, Brian H. Kopell

**Affiliations:** ^1^Department of Psychology and Neuroscience, Duke University, Durham, NC, United States; ^2^SK Life Science, Inc., Paramus, NJ, United States; ^3^Center for Neuromodulation, Department of Neurosurgery, Icahn School of Medicine at Mount Sinai, New York, NY, United States; ^4^Nash Family Center for Advanced Circuit Therapeutics, Icahn School of Medicine at Mount Sinai, New York, NY, United States

**Keywords:** thalamic neuromodulation, deep brain stimulation (DBS), responsive neurostimulation (RNS), central median nucleus of the thalamus, drug-resistant epilepsy (DRE), Holmes tremor

## Introduction

The thalamus is considered the “central forum” of the brain where most principal connections converge and diverge, principally creating the temporal scaffold in which disparate anatomical brain regions are organized into a coherent module subserving behavior. Harnessing this extensive network through neuromodulation has traditionally addressed numerous difficult-to-treat movement disorders such as essential tremor and Parkinson's disease. The therapeutic potential of stimulating this phylogenetically ancient brain structure for other neurological disorders has gained considerable attention since the turn of the 21st century. Even if not stimulated directly, biomarkers, or neural signatures from the thalamus are increasingly being used to assess the therapeutic success of disparate types of neuromodulation technologies. This increased clinical utilization raises the question: Do “all roads” eventually lead to the thalamus for neuromodulation? In this Research Topic of *Frontiers in Human Neuroscience*, we collected a series of articles that highlight the emerging use of thalamic neuromodulation for difficult-to-treat neurological disorders. First, a perspective article by Mendonça et al. argues for the use of neuromodulation targeting the thalamus for uncommon movement disorders. Two review articles then showcase the expanding use of deep brain stimulation (DBS) and Responsive Neurostimulation (RNS) to target discrete nuclei of the thalamus for treating intractable epilepsies (Feigen and Eskandar; Zillgitt et al.). Finally, three original research reports complement these reviews by using both DBS and RNS to control seizures in adult and pediatric patients *via* thalamic stimulation (Agashe et al.; Beaudrault et al.; Roa et al.).

## An argument in favor of deep brain stimulation for uncommon movement disorders

Deep brain stimulation is an established therapy for the management of motor symptoms in patients with medically refractory Parkinson's disease and essential tremor. The current challenge is implementing DBS for movement disorders that may not develop in the context of a degenerative disease, but rather from a static brain lesion. A perspective article by Mendonça et al. argues for the consideration of DBS for medically refractory, post-lesional movement disorders. But how can the effectiveness and risk of DBS be assessed for such rare disorders in the absence of randomized controlled trials? The authors eloquently use Holmes tremor (HT), a post-lesional movement disorder, to illustrate the ethical imperative to consider DBS among heterogeneous patient populations based on an “N-of-1” trial design approach.

Because the ventral thalamus is targeted in more than half of reported DBS cases for HT, the authors use previously reported electrode coordinate information to create a network connectivity map based on simulated volumes of tissue activated (VTA). Using this individualized approach, Mendonça et al. found that ventral thalamic VTAs have significantly higher connectivity with a previously described HT lesion network map than non-thalamic VTAs. In other words, even with dissimilar lesion locations, the ventral thalamus is a candidate neuromodulation network node for HT. The authors conclude that generation of a database through a paired and iterative *N*-of-1 trial approach for uncommon movement disorders will ensure both DBS electrode placement and parameter optimization when combined with patient-specific connectivity mapping of responses.

## Thalamic neuromodulation for intractable epilepsies: A new frontier

The treatment of drug-resistant, intractable epilepsies has entered a new frontier with the use of thalamic neuromodulation (Ilyas et al., 2022). Early targeting of the anterior (ANT) nucleus with DBS used an open-loop stimulation approach to treat several types of intractable epilepsy (Feigen and Eskandar). Now, with the emergence of a closed-loop system, clinicians are evaluating the ability to use thalamic recordings to deliver tailored stimulation. Beaudreault et al. and Roa et al. showcase the utility of the RNS System to reliably detect and treat seizures at several thalamic targets, including the ANT and central median (CM) nuclei. These studies are exciting from a methodological perspective and add to the scant literature on thalamic closed-loop neuromodulation for pediatric epilepsies (Welch et al., 2021). Beaudreault et al. further report the ability to detect focal and generalized seizures with bilateral pulvinar leads, an understudied thalamic target for posterior cortex epilepsies (Burdette et al., 2021; Feigen and Eskandar).

Of note is the off-label use of these stimulation technologies to control seizures by targeting CM: An intralaminar thalamic nucleus that has garnered considerable interest from epileptologists and neurosurgeons alike (Beaudreault et al.; Roa et al.). Agashe et al. substantiates this interest by providing a case report and survey of the literature on CM-DBS for a genetic epilepsy characterized by thalamocortical dysfunction. The authors show that CM-DBS produced a significant reduction in generalized tonic-clonic seizures in a cognitively normal patient. Zillgitt et al. further supports the targeting of CM for idiopathic generalized epilepsy (IGE) by providing an illustrative case using the RNS System. In fact, CM-RNS for the treatment of IGE has led to the initiation of a prospective single blind, multi-center, randomized study (NAUTILUS). The field waits in anticipation for the trial's results as surgical options for genetic generalized epilepsies have historically been limited.

## Do “all roads” lead to the thalamus for neuromodulation?

The thalamus is emblematic of the Roman Empire's *Miliarium Aureum*, a point where all roads were said to eventually converge. The studies herein support this metonymy of a centrally located brain structure with the power to influence far-reaching neural networks. Though a question remains: Is the thalamus a promising stimulation target merely because of its anatomical location, or are off-target effects of its “roads” responsible for observed success? Several recent articles hint at the latter, whether it is stimulation of the mammillothalamic tract for intractable epilepsy (Freund et al., 2022) or the Fields of Forel for Parkinson's disease and dystonia (Horisawa et al., 2021; Watson et al., 2021b). Nevertheless, the emerging insight that many of these pathophysiological states are characterized by temporal activity, which the thalamus is integral in generating and maintaining across brain networks, implicates this region in future neuromodulation strategies.

What can we expect in years to come? The use of thalamic biomarkers to guide therapy decisions, further individualization of thalamic electrode placement through network-based approaches and exploring the therapeutic potential of stimulating other thalamic nuclei, such as the central lateral nucleus for restoring conscious arousal (Kundishora et al., 2017). Time will tell if the underutilization of surgery and an overestimation of its risks will impede these advances (Mendonça et al.; Watson et al., 2021a).

## Author contributions

All authors drafted, revised, and approved final version of the editorial.
